# An Unequal Clustering and Multi-Hop Routing Protocol Based on Fuzzy Logic and Q-Learning in WSNs

**DOI:** 10.3390/e27020118

**Published:** 2025-01-24

**Authors:** Zhen Wang, Jin Duan

**Affiliations:** School of Electronic Information Engineering, Changchun University of Science and Technology, Changchun 130022, China; wangzhen1531002@163.com

**Keywords:** WSNs, fuzzy logic, Q-learning, unequal clustering

## Abstract

Clustering-based routing techniques are key to significantly extending the lifetime of wireless sensor networks (WSNs). However, these approaches often do not address the common hotspot issue in multi-hop WSNs. To overcome this challenge and enhance network lifespan, this study presents FQ-UCR, a hybrid approach that merges unequal clustering based on fuzzy logic (FL) with routing optimized through Q-learning. In FQ-UCR, a tentative CH employs a fuzzy inference system (FIS) to compute its probability of being selected as the final CH. By using the Q-learning algorithm, the best forwarding cluster head (CH) is chosen to construct the data transmission route between the CHs and the base station (BS). The approach is extensively evaluated and compared with protocols like EEUC and CHEF. Simulation results demonstrate that FQ-UCR improves energy efficiency across all nodes, significantly extends network lifetime, and effectively alleviates the hotspot issue.

## 1. Introduction

WSNs typically consist of numerous energy-constrained sensor nodes that are distributed across a designated area. These nodes are generally deployed in a random manner and are capable of sensing environmental data, processing them locally, and transmitting the information to the BS [1,2,3]. WSNs provide substantial capabilities across diverse applications, including in science, the military, healthcare, engineering, environmental monitoring, home automation, area surveillance, and disaster response [4,5,6]. Due to their placement in remote locations, replacing the batteries of sensor nodes is often challenging. These batteries are gradually depleted during the processes of sensing, data aggregation, and transmission. Therefore, finding effective solutions to reduce energy consumption in WSNs continues to be a major challenge [7,8,9].

Clustering is a widely used strategy to improve energy efficiency and extend the lifespan of WSNs [10,11]. In the clustering mechanism, CHs aggregate data from member nodes and transmit them to the destination node, either directly or through intermediate CHs [12,13]. To minimize energy consumption, many clustering protocols employ a multi-hop method where CHs near the BS serve as relays to forward aggregated data from other CHs to the BS. However, these CHs near the BS experience significantly higher traffic loads and energy consumption compared to other nodes, making them more vulnerable to rapid energy depletion and contributing to the hotspot issue within WSNs [14,15]. To overcome this challenge, numerous techniques for unequal clustering have been proposed. These approaches segment the network into clusters of different sizes, positioning smaller ones nearer to the BS and larger ones at a greater distance [16,17].

Clustering and routing protocols are considered NP-hard optimization problems, where optimal or near-optimal solutions can be attained through intelligent computational methods such as fuzzy logic [18] and Q-learning [19]. Fuzzy logic, in particular, excels at handling the uncertainties inherent in clustering and routing, offering greater flexibility than traditional crisp logic. By effectively integrating various input parameters, fuzzy logic helps derive optimal solutions, making it a popular choice for CH selection [18]. In contrast, reinforcement learning (RL) is highly effective for addressing routing challenges in WSNs due to its ability to capture the dynamic nature of the network and environment. In this context, the action of each sensor node involves selecting the next hop to forward sensed data to the sink node. Among RL algorithms, Q-learning, a model-free approach, is widely used in routing problems within sensor networks, enabling adaptive and efficient route optimization [19].

This work addresses the hotspot issue in WSNs by adopting a hybrid strategy that combines fuzzy logic with Q-learning to improve both clustering and routing efficiency. The main contributions of this study, introduced through the FQ-UCR algorithm, can be summarized as follows:

(1) The CH selection probability is calculated using inputs such as residual energy, distance to the BS, the number of neighbors, and node centrality.
(2) The selection of a relay CH is based on maximizing both the Q-value and the associated reward.
(3) The proposed algorithm was simulated to showcase its advantages over existing algorithms, such as the EEUC [20] and CHEF [21] protocols.

The structure of this paper is as follows: Section 2 provides an overview of related research and is followed by Section 3, which presents the fundamental model of the proposed algorithm. Section 4 offers a thorough breakdown of the proposed FQ-UCR algorithm. In Section 5, the simulation results are presented. Finally, Section 6 wraps up the paper and discusses avenues for future exploration.

## 2. Related Works

Recently, several protocols employing unequal clustering have been developed to improve energy efficiency in WSNs. The EEUC [20] protocol uses a probabilistic threshold, *T*, to determine tentative CHs. The final CH is selected by choosing the node with the highest residual energy within the competition radius. During the multi-hop data transmission phase, relay nodes are chosen based on their distance from the BS and remaining energy. The UCR [22] protocol designates CHs based on the residual energy of the nodes, thereby creating smaller clusters in proximity to the BS and larger clusters at greater distances. The EADUC [23] protocol calculates CH candidacy weights by considering both the remaining energy and the node’s degree.

Recent research has indicated that clustering in WSNs is influenced by various interconnected factors. Fuzzy logic effectively manages uncertainties and combines different parameters to obtain the desired results, making it highly effective for clustering in WSNs [24]. The CHEF [21] protocol probabilistically selects tentative CHs and employs a FIS to assess the likelihood of a node being elected as the final CH. This assessment is based on various inputs, including local distance and residual energy. The EAUCF [25] protocol uses an FIS to calculate the competition radius, considering a node’s remaining energy and its distance to the BS. It also probabilistically identifies preliminary CHs. Ultimately, the final CH is selected as the tentative CH with the highest remaining energy within this radius. To identify CHs, the EEFUC [26] protocol utilizes an FIS that evaluates factors such as node energy, distance to the BS, and the number of active nodes. CHs are responsible for data aggregation and multi-hop transmission to the BS. In EFUCA [27], each node can become a CH by making an educated decision using fuzzy logic. In the clustering stage, the fuzzy logic system uses the remaining power, proximity to the base station, and average distance to communicating nodes as inputs to determine the rank and competition radius. Another fuzzy logic system, whose inputs are the next-hop rank, proximity to the next hop, and distance reduced to the BS, is used to finish the next-hop selection for effective data forwarding in order to further extend the network lifetime. The FMCB-ER [28] protocol was introduced as a hierarchical routing approach designed to improve network longevity. It combines Bio-inspired Energy-efficient Routing with Fuzzy Multi-Criteria Clustering techniques. The protocol employs a grid-based clustering strategy to form robust clusters, leveraging adaptive fuzzy multi-criteria decision-making, which integrates TOPSIS and fuzzy AHP for optimal CH selection, along with the Emperor Penguin Optimization (EPO) algorithm to determine the most effective routing path for data transmission from CHs to the sink. Singh [29] used the fuzzy grey wolf optimization (F-GWO) algorithm for cluster head selection as part of an energy-efficient clustering and routing protocol for a WSN. The opportunistic routing method, which minimizes power consumption and balances energy consumption among nodes in WSNs, was used, and the fuzzy parameters were evaluated with an algorithm that produced an enhanced route. However, the implemented algorithm did not take into account the fitness function of energy and distance that caused packet drop in the network. The Fuzzy Multi-Criteria Clustering and Bio-inspired Energy-efficient Routing (FMCB-ER) [30] protocol was presented as a hierarchical routing technique to increase the operational lifetime of networks. It uses a grid-based clustering strategy to create strong clusters and adaptive fuzzy multi-criteria decision-making, which combines TOPSIS and fuzzy AHP for the best CH selection and the Emperor Penguin Optimization (EPO) algorithm to determine the most effective routing path for data transfer from CHs to the sink.

As research has progressed, it has become evident that WSNs often operate in rapidly changing environments, requiring the development of routing algorithms that can quickly adapt and make decisions. Reinforcement learning (RL) aims to maximize an agent’s reward by taking a series of actions based on feedback from a dynamic environment. RL is particularly well suited for WSN routing as it can effectively adjust to the network’s evolving conditions [31]. Yun et al. [19] introduced an innovative routing algorithm that leverages Q-learning to enhance energy efficiency while incorporating data aggregation awareness. This strategy effectively cuts down on redundant data transmissions, contributing to a longer network lifespan. Nevertheless, it does not adequately tackle the challenge of reducing delays in data aggregation. Su et al. [32] offer a method for extending network lifetime and improving energy efficiency in WSNs through Q-learning-based routing. By taking into account different energy consumption criteria, it enables nodes to choose nearby nodes for transmission, leading to a more balanced and less energy-consuming process. Yao et al. [33] proposed MRP-ICHI, a multi-hop clustering routing protocol that combines an improved Coronavirus Herd Immunity Optimizer (CHIO) with Q-learning to enhance network performance.The improved CHIO algorithm optimizes network clustering by formulating a fitness function that incorporates node energy levels and location attributes. To ensure efficient data transmission, the protocol also utilizes Q-learning to establish an effective multi-hop routing mechanism between CHs and the BS. Using grey wolf optimization and Q-learning, Bedi et al. [34] developed an innovative routing system for wireless body area networks (WBANs). By optimizing the network lifetime through these techniques, the protocol adjusts routing paths in response to energy depletion in various network nodes, increasing energy efficiency.

## 3. System Model

### 3.1. Network Model

The assumptions underlying the design of the proposed FQ-UCR are outlined as follows:

(1) A total of *N* sensor nodes are dispersed randomly across an M×M region.
(2) All sensor nodes are initialized with the same amount of energy upon deployment.
(3) Once deployed, it is assumed that both the sensor nodes and the BS stay in fixed positions.
(4) The sensor nodes operate on a limited energy supply, while the BS has an unlimited energy source.

**Figure 1:**
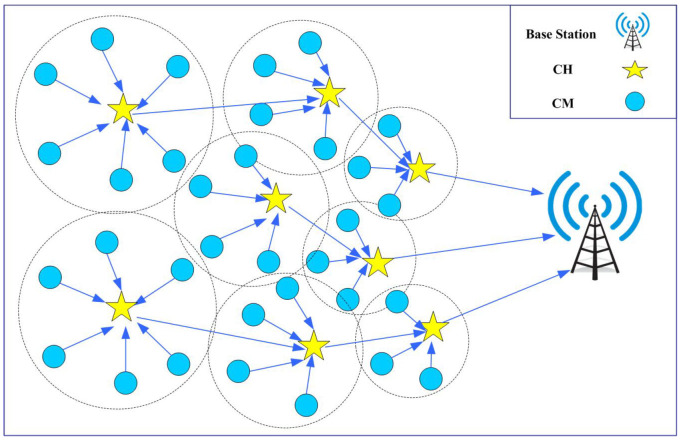
The network model employed in this study.

The network model of the proposed FQ-UCR is shown in Figure 1.

### 3.2. Energy Model

**Figure 2:**
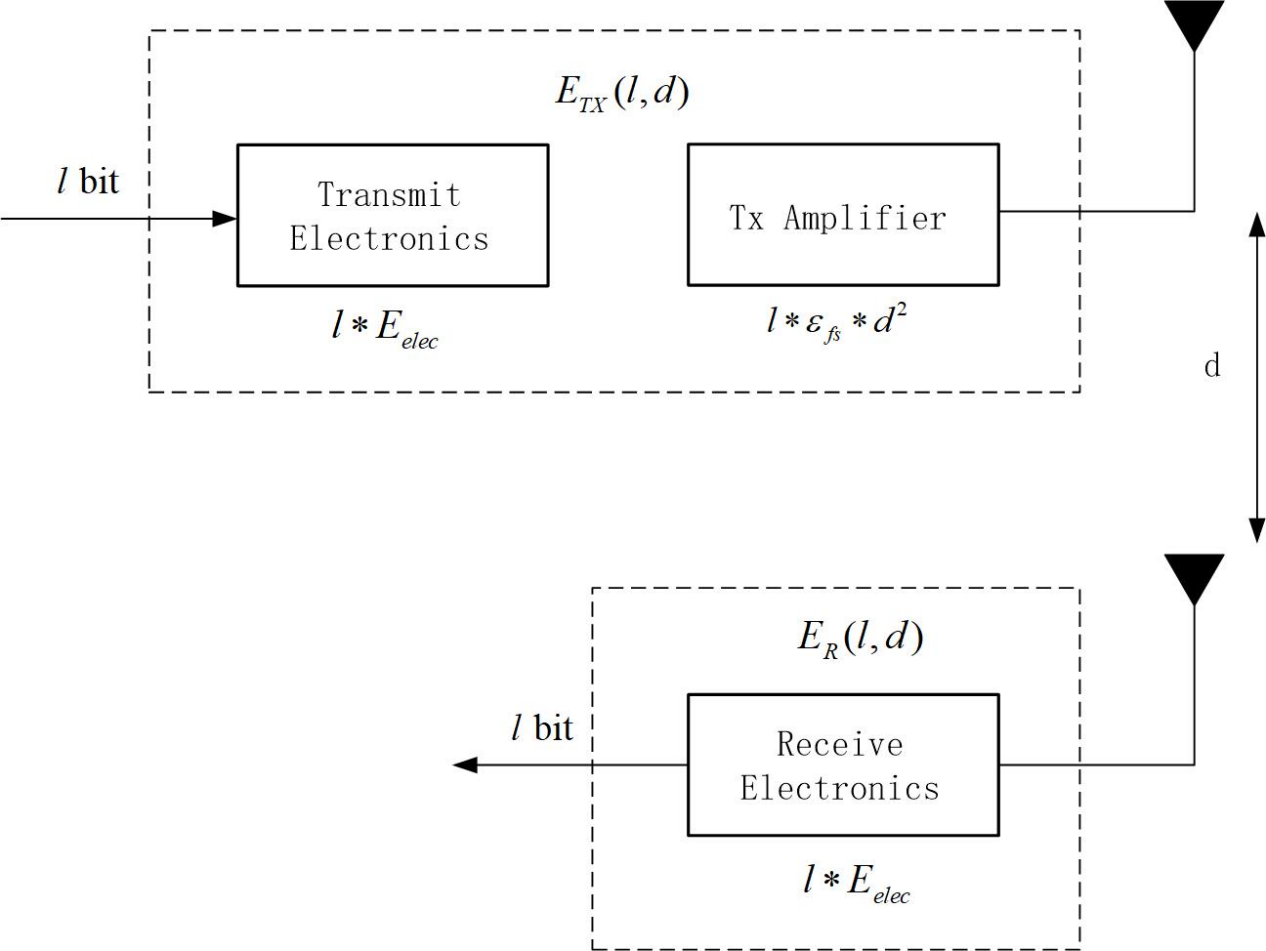
Radio model.

In FQ-UCR, the radio model from [35] is utilized, as depicted in Figure 2. The power consumption model for transmitting *l*-bit data packets from the transmitter to the receiver, separated by the distance *d*, is calculated by Equations (1) and the energy consumption required to obtain *l*-bit of data by Equations (2):

(1) $$E_{TX}\left(l,d\right)=\left\{\begin{array}{cc} l\times E_{elec}+l\times \varepsilon_{fs}\times d^{2} & \;if\;\;d\leq d_{0}\\ l\times E_{elec}+l\times \varepsilon_{mp}\times d^{4} & \;if\;\;d>d_{0} \end{array}\right.$$

(2) $$E_{RX}\left(l\right)=l\times E_{elec}$$

The threshold $d_{0}$ identifies whether the free-space model $\epsilon_{fs}$ or the multipath model $\epsilon_{mp}$ applies, with $E_{elec}$ signifying the electronic circuitry’s energy consumption. The calculation for $d_{0}$ is expressed by the equation below:

(3) $$d_{0}=\sqrt{\frac{\epsilon_{fs}}{\epsilon_{mp}}}$$

The multipath fading model is applied when $d>d_{0}$, where the transmit energy usage $E_{TX}\left(l,d\right)$ follows a quadratic relationship with *d*. Conversely, the free-space model is used when $d\leq d_{0}$. Therefore, when designing cluster routing algorithms, it is typically necessary to ensure that the transmission distance *d* between nodes does not exceed $d_{0}$.

## 4. Proposed Method

The FQ-UCR protocol employs an unequal clustering technique, which is executed in two major phases: clustering and data transmission. During the clustering phase, the algorithm leverages an FIS to determine the probability of nodes becoming CHs and their competition radius, prioritizing nodes with higher probabilities for CH selection. In the data transmission phase, Q-learning optimizes the multi-hop routing process, with relay CHs being chosen based on their updated Q-values to relay data to the BS.

**Figure 3:**
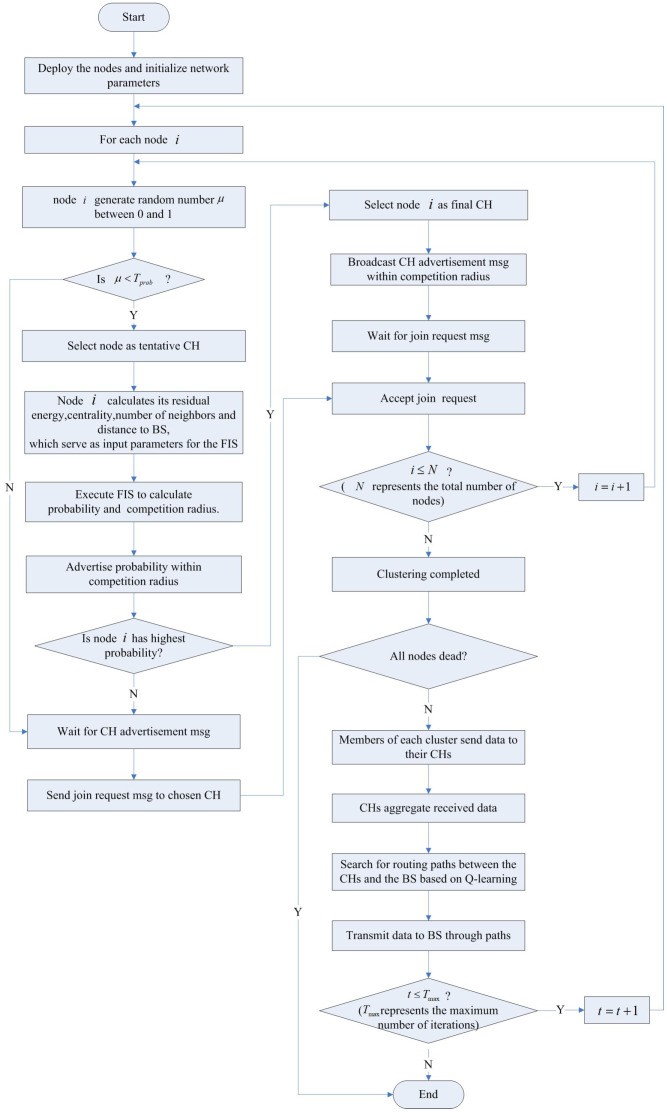
Flow chart of proposed FQ-UCR protocol.

Figure 3 presents a flowchart that outlines the steps of the proposed method.

### 4.1. Fuzzy Logic Model

**Figure 4:**
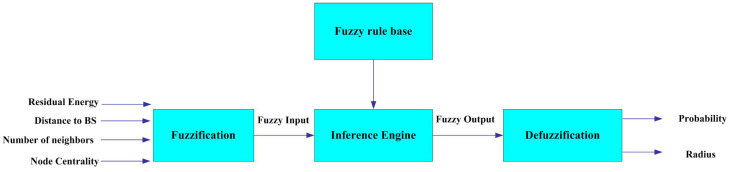
Block diagram of FIS.

The FQ-UCR protocol uses an FL approach for the selection of CHs, as depicted in Figure 4, and is comprised of four main steps [36].

**Fuzzification:** this transforms the clear input into the relevant language variable.

**Fuzzy rule base:** this holds a collection of IF-THEN rules that govern decision- making processes.

**Fuzzy inference engine:** this utilizes fuzzy rules to analyze input data and generate outputs.

**Defuzzification:** this transforms fuzzy outputs into numbers.

### 4.2. Fuzzy Logic-Based Clustering

**Figure 5:**
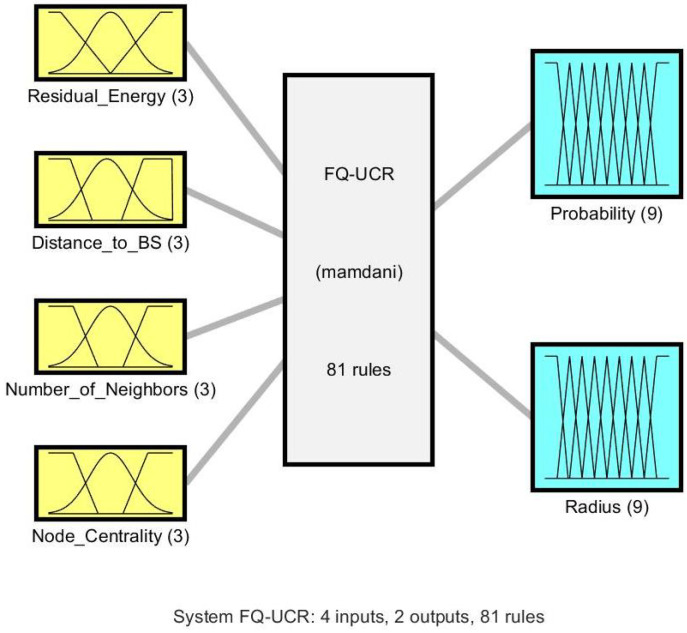
FIS developed for selecting CHs in FQ-UCR.

In each clustering round, each sensor node generates a random number between 0 and 1. If the generated random number is less than the threshold *T*, the node is selected as a tentative CH. As depicted in Figure 5, once the potential CHs are determined, they use a predefined FIS to evaluate the probability of becoming the final CH and calculate their competition radius.

The input variables in FQ-UCR include the residual energy, distance to the BS, number of neighbors, and node centrality.

**Residual Energy (RE):**

This factor is crucial as sensor nodes with higher remaining energy have a greater likelihood of being chosen as CHs. The remaining energy of a node is calculated using Formula (6).

(4) $$RE=\frac{E_{ini}-E_{con}\left(i\right)}{E_{ini}}$$

(5) $$E_{con}\left(i\right)=\left\{\begin{array}{c} E_{non-CH},\;\mathrm{if}\;\mathrm{node}\;i\;\mathrm{is}\;\mathrm{the}\;\mathrm{normal}\;\mathrm{node}\\ E_{CH},\;\mathrm{if}\;\mathrm{node}\;i\;\mathrm{is}\;\mathrm{the}\;\mathrm{CH} \end{array}\right.$$

In this context, $E_{ini}$ indicates the initial energy assigned to a node, whereas $E_{con}\left(i\right)$ represents the amount of energy utilized by the node *i*.

The communication cost of a CH is denoted as $E_{CH}$ and is determined using the following equation:

(6) $$E_{CH}=k\times l\times E_{elec}+k\times l\times \varepsilon_{fs}\times d_{toBS}^{2}+E_{DA}$$

(7) $$E_{DA}=k\times l\times E_{pDb}$$

where $d_{toBS}$ is the distance from the CH to the next hop or BS, $E_{DA}$ is the energy for data aggregation, *l* is the number of bits, *k* is the cluster size, and $E_{pDb}$ is the energy for 1-bit fusion.

The energy consumed by a cluster member node, denoted as $E_{non-CH}$, denotes the energy consumed by a cluster member node and is calculated by

(8) $$E_{non-CH}=l\times \left(E_{elec}+\varepsilon_{fs}d_{toCH}^{2}\right)$$

In this context, $d_{toCH}$ represents the distance between a cluster member and its corresponding CH.

**Figure 6:**
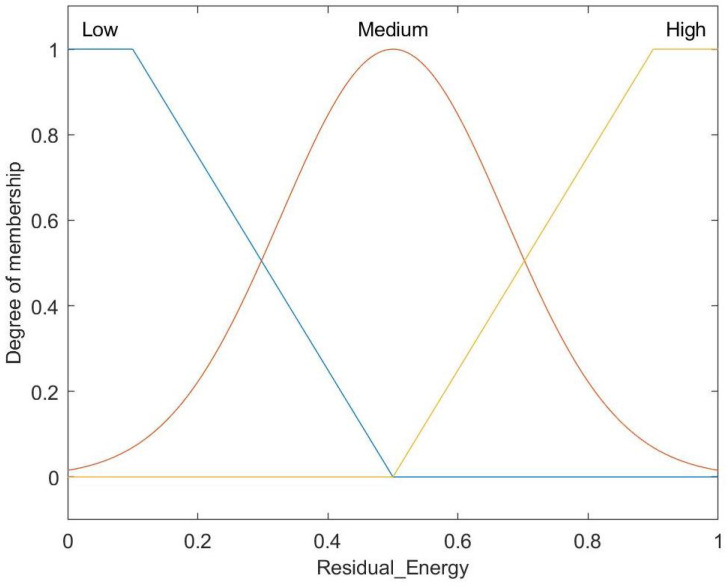
Membership function for residual energy.

Figure 6 presents the fuzzy set for the residual energy input, categorized into Low, Medium, and High levels. The Low and High categories are defined by trapezoidal membership functions, whereas the Medium category is characterized by a Gaussian membership function.

**Distance to BS (DisToBs):**

This factor is vital because a shorter distance between the CH and BS leads to reduced energy consumption during communication. The distance to the BS is calculated using Formula (8).

(9) $$DisToBs=\sqrt{{\left(x_{i}-X_{BS}\right)}^{2}+{\left(y_{i}-Y_{BS}\right)}^{2}}$$

where $\left(x_{i},y_{i}\right)$ and $\left(X_{BS},Y_{BS}\right)$ denote the position of the CH and the BS.

**Figure 7:**
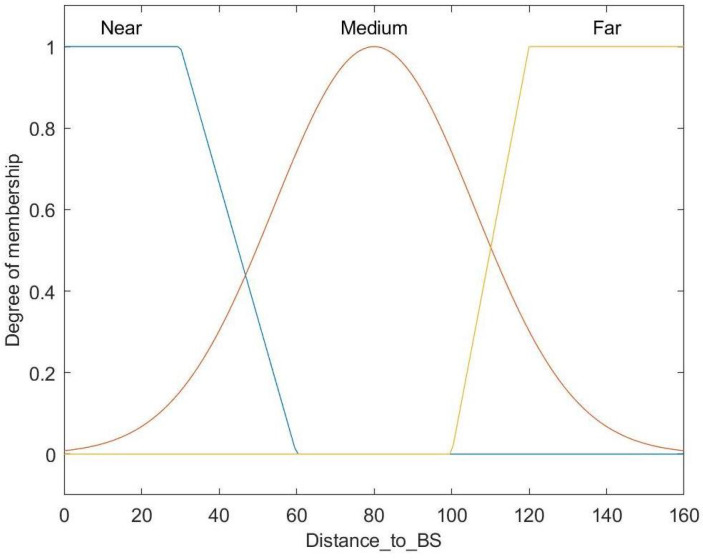
Membership function for distance to base.

Figure 7 shows the fuzzy set for the distance to the BS, which is classified into three ranges: Near, Medium, and Far. The Near and Far ranges are represented using trapezoidal membership functions, whereas a Gaussian membership function is used for the Medium range.

**Number of neighbors (NN):** This factor represents the count of neighboring nodes that have energy levels above the average. A higher number of such neighbors improves data transmission efficiency and reduces the data forwarding load on each neighbor node of the cluster head, thus promoting overall system energy optimization. The calculation of the NN is outlined in Equations (9) and (10).

(10) $$NN=\frac{Num\left(E_{res}\left(Neighbor\left(i\right)\right)>E_{avg}\right)}{totalAlive}$$

where Num(Neighbor(i)) denotes the number of neighbor nodes of the node *i*, and totalAlive represents the total number of active nodes in the network.

**Figure 8:**
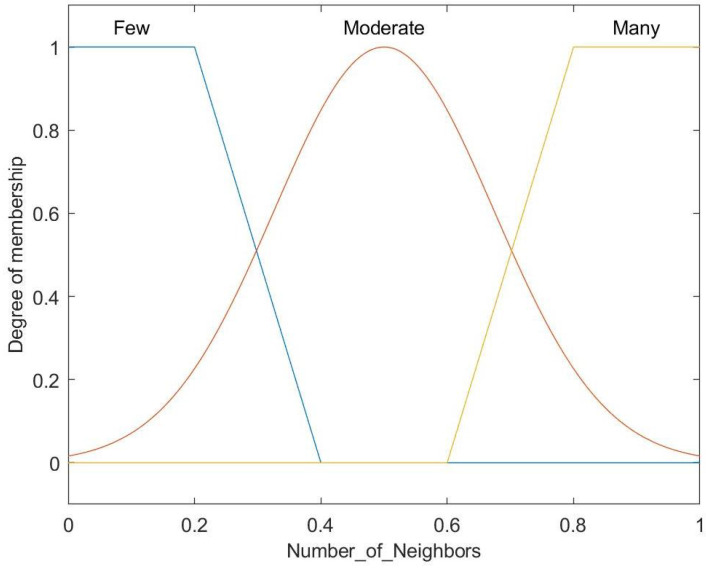
Membership function for number of neighbors.

Figure 8 illustrates the fuzzy set for the input variable of the number of neighbors, with three categories: Few, Moderate, and Many. Trapezoidal membership functions define Few and Many, while the Moderate category is represented by a Gaussian membership function.

**Node Centrality (NC):**

This factor reflects a node’s central position among its neighbors. Centrality evaluates a node’s position within its cluster, reflecting how evenly it is spaced relative to other cluster members. A CH at the edge uses more energy due to its greater distance from other nodes. Equation (12) outlines the calculation of a node’s centrality.

(11) $$NC=\left\{\begin{array}{c} 1,Num(Neighbor(i))=0\\ \frac{\sqrt{\frac{\sum_{j=1}^{Num(Neighbor(i))}dis^{2}\left(i,j\right)}{Num(Neighbor(i))}}}{Dimension},Num\left(Neighbor\left(i\right)\right)>0 \end{array}\right.$$

where $dis^{2}\left(i,j\right)$ denotes the distance between the nodes *i* and *j* and the Dimension value is *A* in the A×A field area.

**Figure 9:**
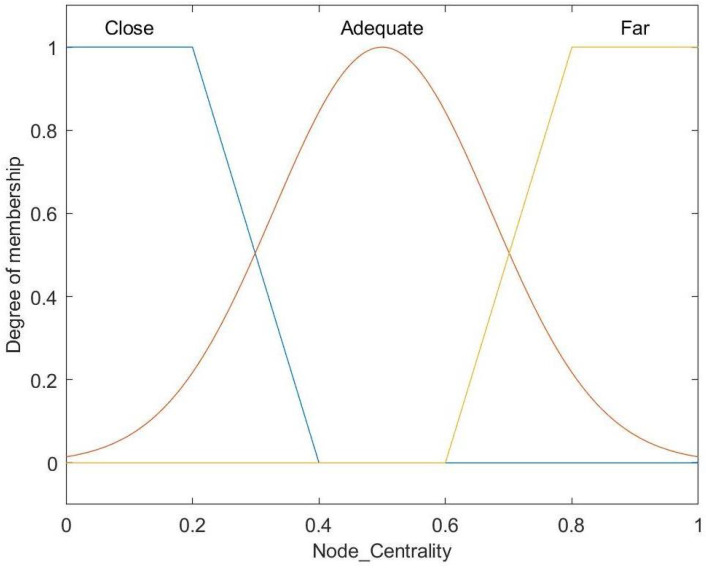
Membership function for node centrality.

Figure 9 illustrates the fuzzy set for the input variable of a node’s centrality, consisting of three linguistic categories: Close, Adequate, and Far. Close and Far are represented by trapezoidal membership functions, while Adequate is modeled with a Gaussian membership function.

FQ-UCR has two output variables: the CH probability and competition radius.

**CH probability:** this probability is calculated to select the optimal CH among sensor nodes, with nodes having higher probabilities being chosen as CHs.

**Figure 10:**
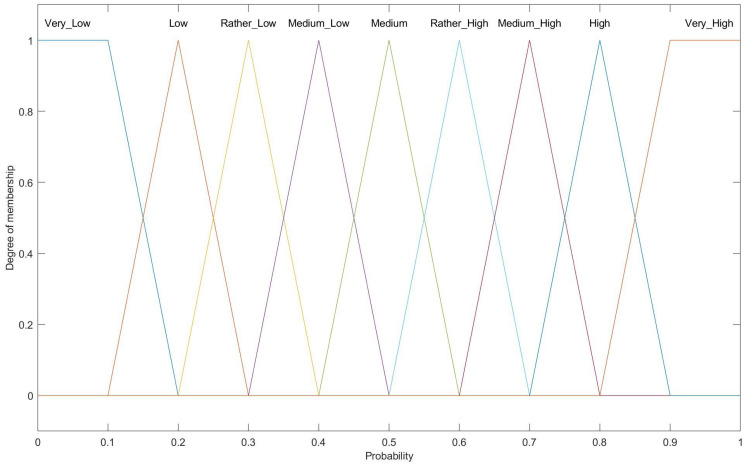
Membership function for output variables for CH selection (probability).

Figure 10 depicts the fuzzy output variable for probability, which includes nine linguistic terms: Very Low, Low, Rather Low, Medium–Low, Medium, Medium–High, Rather High, High, and Very High. Triangular membership functions are used for the majority of these terms.

**Competition radius:** this refers to the broadcast range and competition area of a tentative CH, with a larger radius raising the likelihood of selection.

**Figure 11:**
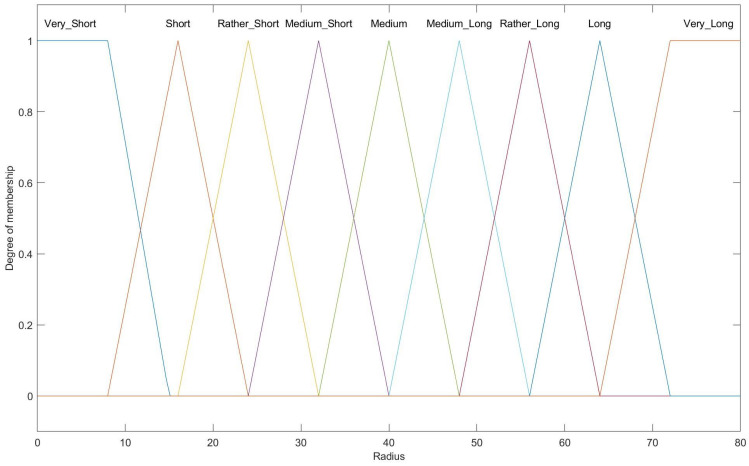
Membership function for output variables for radius.

Figure 11 presents the fuzzy set for the competition radius output variable, which includes nine linguistic variables: Very Short, Short, Rather Short, Medium–Short, Medium, Medium–Long, Rather Long, Long, and Very Long, with most represented by triangular membership functions.

Each sensor node derives the input variables for the fuzzy system using Equations (3)–(11) and translates them into linguistic variables through fuzzification. As outlined in Table 1, these variables are processed using a Mamdani-based fuzzy “if-then” rule set, comprising 3×3×3×3=81 rules in total. To derive precise results, the defuzzification process employs the Center of Area (COA) technique.

Each node computes its CH probability and competition radius and then broadcasts the probability. The node exhibiting the greatest probability is selected as the CH.

Once the CH is elected, it broadcasts “FINAL_CH_MSG” to inform ordinary nodes of its status. Ordinary nodes respond with “JOIN_MSG” to establish their connection to the CH. Algorithm Section 4.2 outlines the detailed selection process.

**Algorithm 1** CH selection in FQ-UCR.
  1:CH_List ← {⌀}  2:S_FQUCR(i).Type ← Normal  3:S_FQUCR(i).TentativeCH ← FALSE  4:**for** i=1 to TN **do**  5: /*TN is the total number of all nodes in the network*/  6: S_FQUCR(i).μ ← rand(0,1)  7: **if** S_FQUCR(i).μ < *T* **then**  8:  /*T ← probability to become tentative CH*/  9:  S_FQUCR(i).TentativeCH ← TRUE10:  Calculate S_FQUCR(i).RE according to Equations (3)–(7) /*S_FQUCR(i).RE is the Residual Energy of node i*/11:  Calculate S_FQUCR(i).DisToBs according to Equation (8) /*S_FQUCR(i).DisToBs is the Distance to BS of node i*/12:  Calculate S_FQUCR(i).NN according to Equations (9) and (10) /*S_FQUCR(i).NN is the Neighbors of node i*/13:  Calculate S_FQUCR(i).NC according to Equation (11) /*S_FQUCR(i).NC is the Centrality of node i*/14:  Fitness ← EvaluateFIS(S_FQUCR(i).RE,S_FQUCR(i).DisToBs,15:  S_FQUCR(i).NN,16:  S_FQUCR(i).NC)17:  S_FQUCR(i).Probability ← Fitness(1)18:  S_FQUCR(i).Radius ← Fitness(2)19:  Broadcast CH_Message(i,S_FQUCR(i).Probability,S_FQUCR(i).Radius)  /*S_FQUCR(i).Probability is the probability to become real CH and S_FQUCR(i).Radius is the Competition radius of node i*/20:  i=i+121: **end if**22:
**end for**
23:**for** i=1 to TN **do**24: **if** S_FQUCR(i).TentativeCH==TRUE **then**25:  **for** j=1 to TN **do**26:   dist(i,j) ← Calculate the distance between node *i* and node *j*27:   **if** dist(i,j)<S_FQUCR(i).Radius **then**28:    **if** S_FQUCR(j).TentativeCH==TRUE **then**29:     **if** S_FQUCR(i).Probability>S_FQUCR(j).Probability **then**30:      S_FQUCR(i).Type ← CH31:      CH_List ← CH_List ∪ {i}32:     **end if**33:    **end if**34:   **end if**35:   j=j+136:  **end for**37: **end if**38: i=i+139:
**end for**



### 4.3. Q-Learning-Based Inter-Cluster Routing

**Figure 12:**
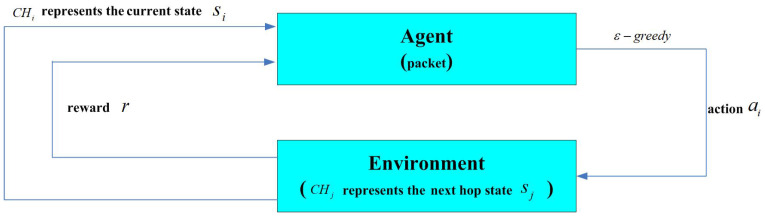
Block diagram of Q-learning.

After the clustering phase, the next task is to establish the optimal transmission path for data to travel from CHs to BS. This routing process can be modeled using finite Markov decision processes and addressed with Q-learning [37]. Q-learning focuses on determining the optimal action strategy within these processes, comprising elements such as the agent, states, actions, rewards, the Q-table, and the environment. Figure 12 presents the Q-learning model employed in this study, demonstrating the approach for optimizing path selection.

(1) The agent (packet) in the current state $s_{i}$ (represented as $CH_{i}$) uses the ϵ-greedy strategy to choose the action $a_{i}$
(2) After executing the action $a_{i}$, the agent (packet) transitions to a new state, $s_{j}$ (represented as $CH_{j}$), and receives an observed reward, $r_{S_{i}S_{j}}^{a_{i}}$. The reward value is
  (12) $$\begin{array}{c} r_{S_{i}S_{j}}^{a_{i}}=\left\{\begin{array}{cc} \frac{E_{res}\left(s_{j}\right)}{\frac{\sum_{j=1}^{k}d\left(s_{i},s_{j}\right)}{k}+d\left(s_{j},BS\right)} & if\;s_{j}\;\mathrm{is}\;\mathrm{not}\;\mathrm{BS}\\ R_{BS} & el\mathrm{se} \end{array}\right. \end{array}$$
  where $E_{res}\left(s_{j}\right)$ is the residual energy of $CH_{j}$, *k* represents the number of neighbor CHs, $d(s_{i},s_{j})$ is the distance between $CH_{i}$ and $CH_{j}$, and $d(s_{j},BS)$ is the distance between $CH_{j}$ and BS.
(3) $s_{j}$ (represented as $CH_{j}$) obtains $\max_{a_{j}\in A_{j}}Q\left(s_{j},a_{j}\right)$. Note that $\max_{a_{j}\in A_{j}}Q_{t}\left(s_{j},a_{j}\right)$ refers to the highest Q-value among all actions available for the state $s_{j}$, where $A_{j}$ is the action set of the state $s_{j}$.
(4) $s_{i}$ (represented as $CH_{i}$) updates $Q\left(s_{i},a_{i}\right)$ using Equation (12).
  (13) $$\begin{array}{c} \begin{array}{cc} Q\left(s_{i},a_{i}\right) & ⟵\left(1-\alpha \right)Q\left(s_{i},a_{i}\right)\\ & +\alpha \left[r_{s_{i}s_{j}}^{a_{i}}+\gamma \max_{a_{j}\in A_{j}}Q\left(s_{j},a_{j}\right)\right] \end{array} \end{array}$$

The multi-hop routing process is outlined in Algorithm Section 4.3.

**Algorithm 2** Q-learning for routing.
**Input:** CHs set $CH\_List=\left\{{CH}_{1},{CH}_{2},{CH}_{3},\cdots ,{CH}_{m}\right\}$**Output:** Route *R* from ${CH}_{i}$ to BS (base station)  1:Initialize and each CH constructs its Q-table  2:$s_{i}$ ← A CH is randomly selected from the CH_List /* $s_{i}$ is the current state */  3:**for** each episode **do**  4: *R* ← *R* ∪ $\left\{s_{i}\right\}$  5: **repeat**  6:  **if** $N_{s_{i}}$ ≠ {⌀} **then**  7:   /* $N_{s_{i}}$ is the next hop set of CH $s_{i}$ */  8:   generate random number *r*  9:   **if** r<ϵ **then**10:    $s_{i}$ selects action $a_{i}$ that has the maximum Q-value.11:   **else**12:    $s_{i}$ randomly selects action $a_{i}$13:   **end if**/*Select action $a_{i}$ using ϵ−greedy strategy,$a_{i}\in N_{s_{i}}$*/14:  **end if**15:  /*Once the action $a_{i}$ is performed, the agent’s state will change from $s_{i}$ to $s_{j}$*/16:  $s_{j}$ obtains $\max_{a_{j}\in A_{j}}Q\left(s_{j},a_{j}\right)$  Calculate reward $r_{s_{i}s_{j}}^{a_{i}}$ using Equation (12);17:  $s_{j}$ feedback $r_{s_{i}s_{j}}^{a_{i}}$ and $\max_{a_{j}\in A_{j}}Q\left(s_{j},a_{j}\right)$ to $s_{i}$;18:  $s_{i}$ Update $Q\left(s_{i},a_{i}\right)$ using Equation (13);19:  $s_{i}=s_{j}$20: **until** $s_{j}==BS$21:
**end for**



## 5. Results and Discussion

### 5.1. Simulation Setup

**Figure 13:**
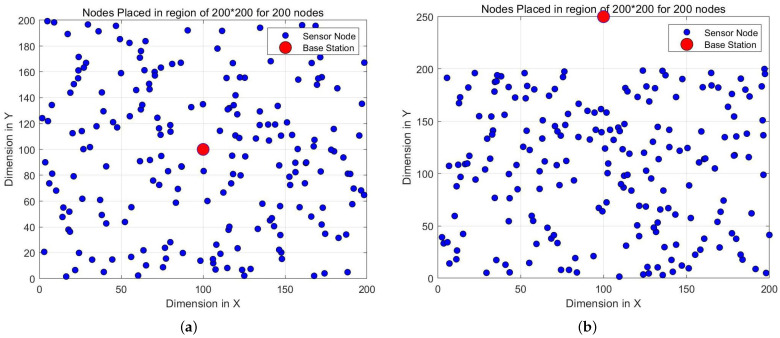
(**a**) Node distribution for Scenario 1. (**b**) Node distribution for Scenario 2.

The proposed protocol was implemented in MATLAB R2020b and compared with the CHEF and EEUC algorithms. It was evaluated in two scenarios, each with 100, 200, and 400 sensor nodes. In Scenario 1, the BS was positioned at the network’s center, while in Scenario 2, it was located outside the network. Initial parameters are provided in Table 2. Figure 13a,b illustrate the configurations for Scenario 1 and Scenario 2, respectively.

### 5.2. Performance Evaluation in Scenario 1

#### 5.2.1. Residual Energy

**Figure 14:**
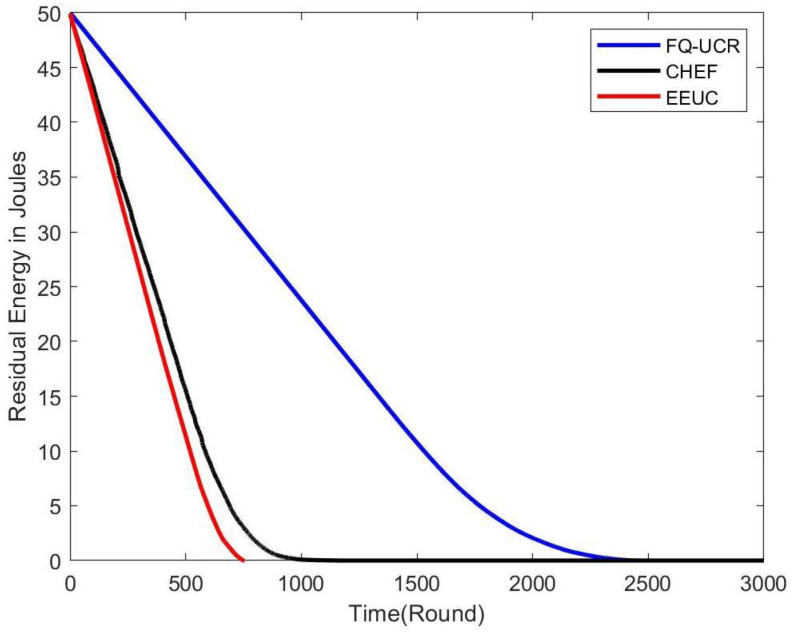
The figure compares the residual energy (in Joules) of 100 nodes, with the BS located at (100, 100), across different protocols.

**Figure 15:**
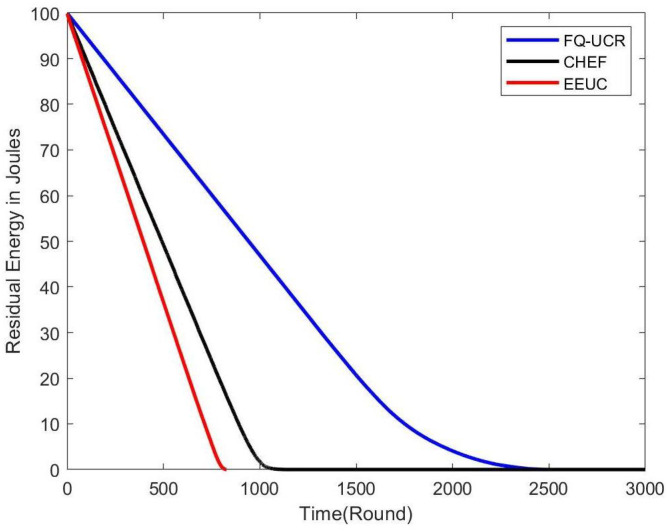
The figure compares the residual energy (in Joules) of 200 nodes, with the BS located at (100, 100), across different protocols.

**Figure 16:**
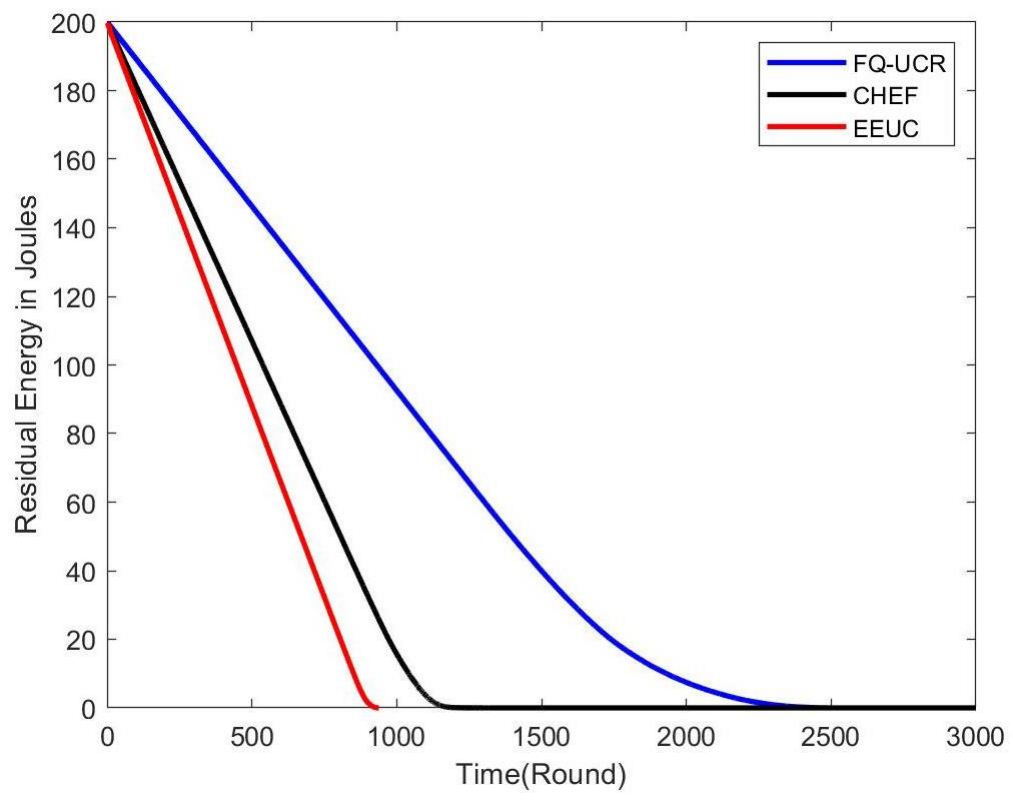
The figure compares the residual energy (in Joules) of 400 nodes, with the BS located at (100, 100), across different protocols.

Figure 14, Figure 15 and Figure 16 present comparisons of residual energy across different protocols at various network densities. As seen in Figure 12, with 100 nodes, the CHEF and EEUC protocols experienced near energy depletion around the 780th round. In contrast, FQ-UCR retained 40.96% of its energy and continued operating until the 2477th round. Figure 13 shows the residual energy performance with 200 nodes, where EEUC and CHEF depleted their energy around round 950. However, FQ-UCR still had 50.43% of the remaining energy. Figure 14 displays results for the 400-node experiment, with EEUC and CHEF running out of energy around the 1110th round, while FQ-UCR operated beyond the 2499th round. This advantage became more pronounced as the node count increased. Overall, FQ-UCR outperformed other protocols in energy efficiency, leading to a longer network lifespan and improved overall performance.

#### 5.2.2. Network Lifetime

**Figure 17:**
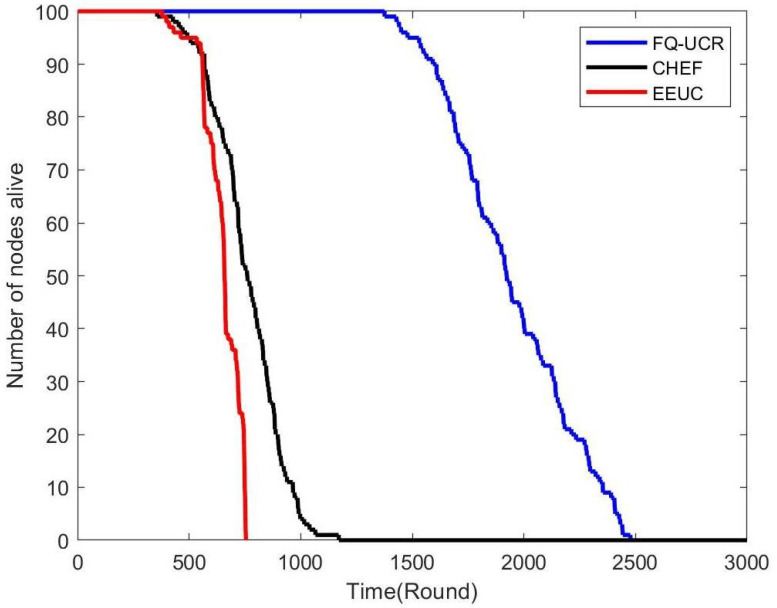
Network lifetime comparison for 100 nodes in Scenario 1.

**Figure 18:**
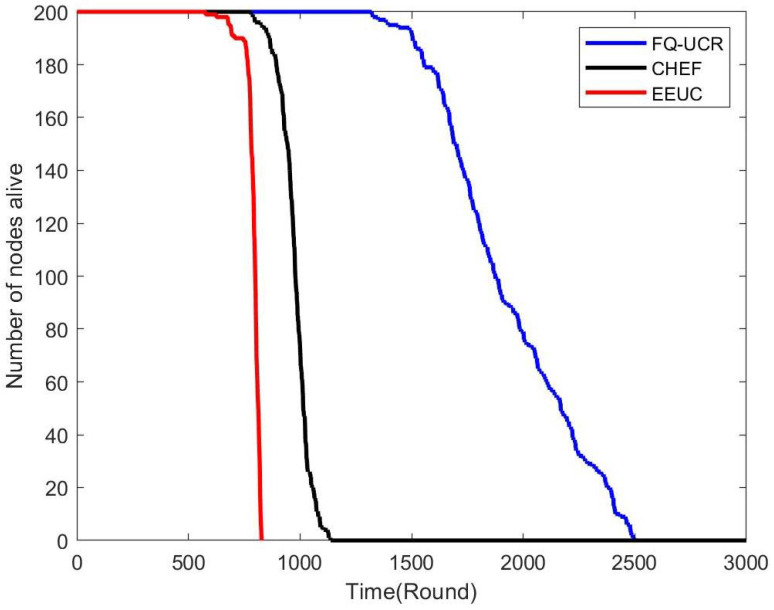
Network lifetime comparison for 200 nodes in Scenario 1.

**Figure 19:**
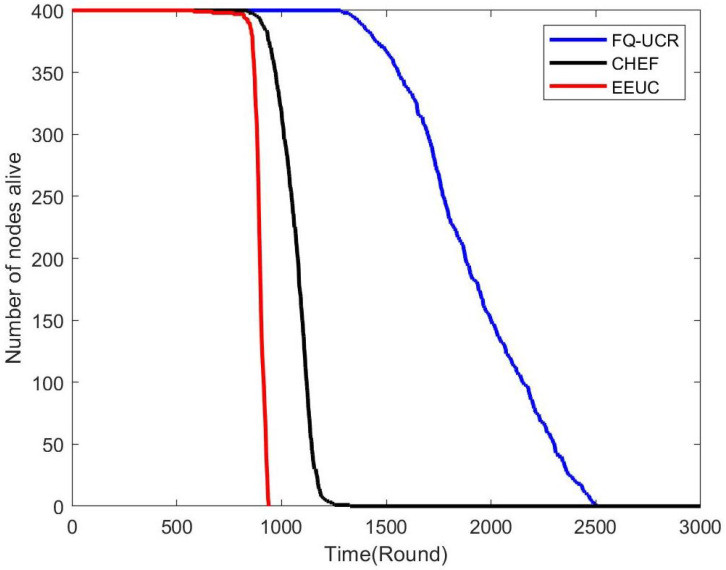
Network lifetime comparison for 400 nodes in Scenario 1.

Figure 17, Figure 18 and Figure 19 present a comparison of the network lifecycle performance of EEUC, CHEF, and FQ-UCR in Scenario 1. For the 100-node network, EEUC resulted in the shortest lifetime, while CHEF attempted to improve this but still underperformed. FQ-UCR, however, achieved the longest network lifetime. With 200 nodes, FQ-UCR surpassed both EEUC and CHEF in network lifetime. For the 400-node scenario, FQ-UCR extended the network lifetime to 2499 rounds, while CHEF and EEUC reached 1326 and 938 rounds, respectively. FQ-UCR clearly surpassed the other two protocols in network lifetime across varying node counts.

#### 5.2.3. Stability Period

**Figure 20:**
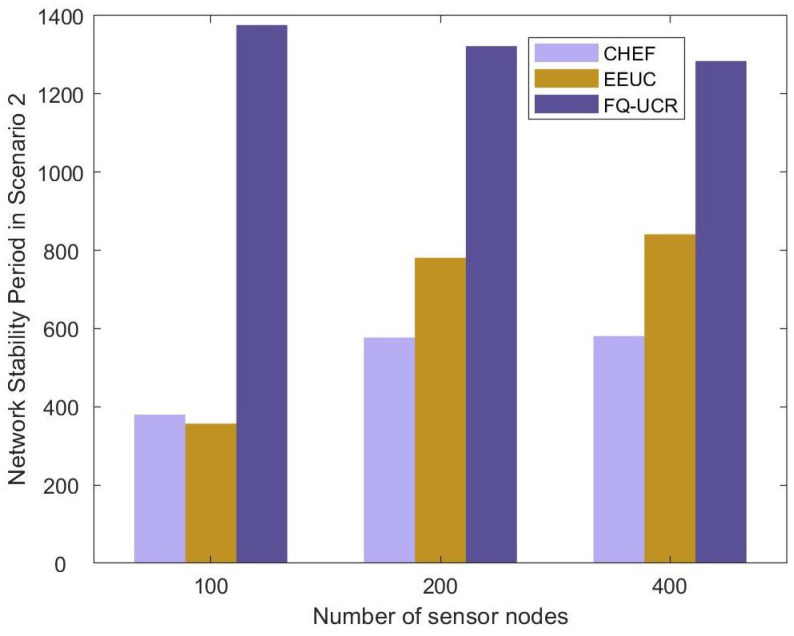
Comparative analysis based on network stability period with BS located at (100, 250).

Table 3 and Figure 20 show the network stability period results for EEUC, CHEF, and FQ-UCR. The proposed protocol stood out with a significant improvement in stability. For the network with 100 nodes, the stability period was 380 rounds for EEUC, 357 rounds for CHEF, and 805 rounds for FQ-UCR, which was the longest. FQ-UCR surpassed the others, offering a 262.1% increase over EEUC and a 285.4% increase over CHEF. As the node count grew to 200 and 400, FQ-UCR continued to lead in stability. These results demonstrate the FQ-UCR algorithm’s effectiveness in prolonging network stability, thereby enhancing overall performance.

#### 5.2.4. Throughput

**Figure 21:**
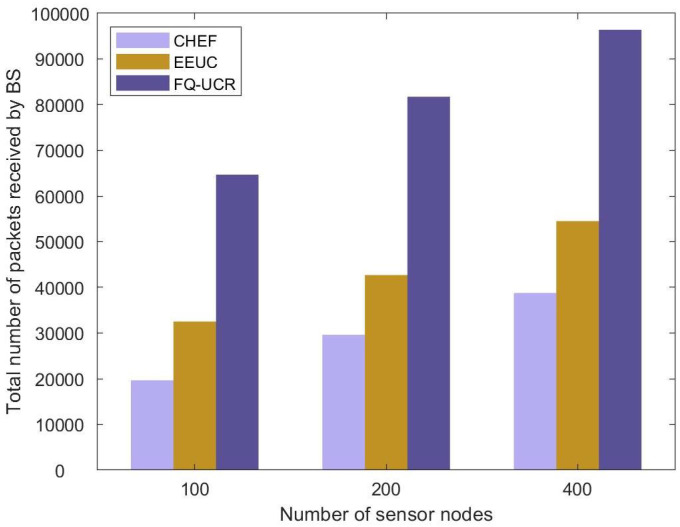
Comparative analysis based on throughput in Scenario 1.

Figure 21 presents a comparative analysis of network throughput for the EEUC, CHEF, and FQ-UCR protocols across different node counts. As illustrated in Figure 21, the proposed FQ-UCR protocol demonstrated a substantial performance advantage over the other protocols. The improvement was due to fuzzy logic clustering and Q-learning routing, which optimized inter-cluster transmission and CH selection, resulting in at least an 98.8% increase in network throughput across various sizes.

### 5.3. Performance Evaluation in Scenario 2

#### 5.3.1. Residual Energy

**Figure 22:**
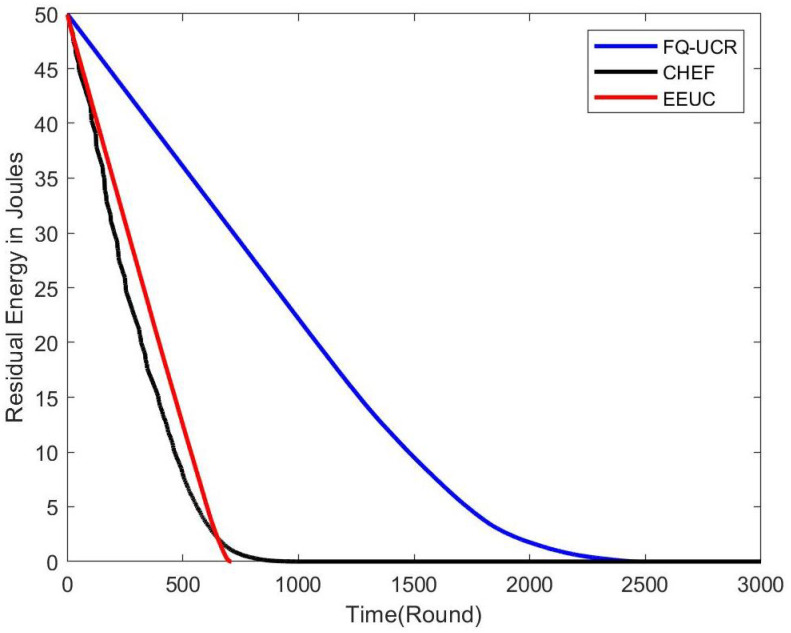
The figure compares the residual energy (in Joules) of 100 nodes, with the BS located at (100, 250), across different protocols.

**Figure 23:**
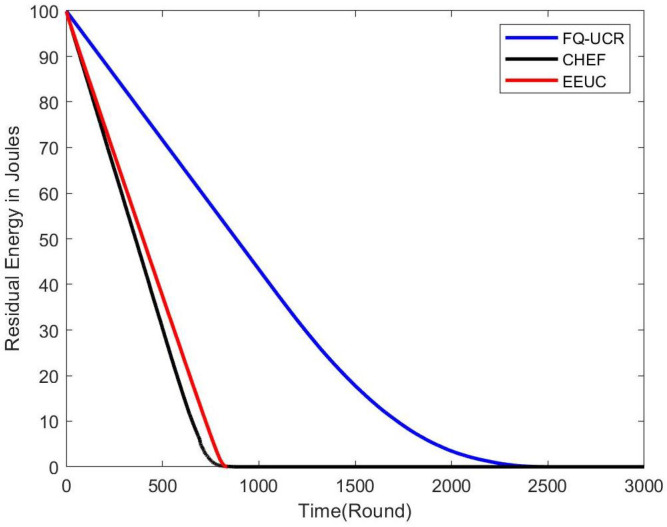
The figure compares the residual energy (in Joules) of 200 nodes, with the BS located at (100, 250), across different protocols.

**Figure 24:**
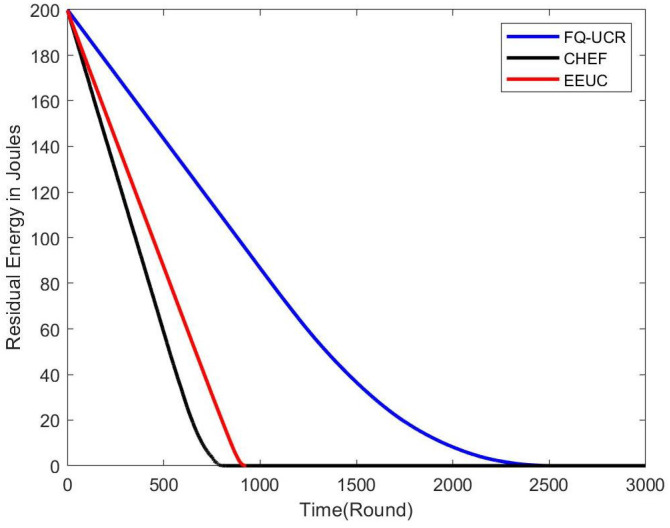
The figure compares the residual energy (in Joules) of 400 nodes, with the BS located at (100, 250), across different protocols.

Figure 22, Figure 23 and Figure 24 illustrate the comparison of the network residual energy among the EEUC, CHEF, and FQ-UCR protocols across different node counts. As depicted in Figure 22, both the EEUC and CHEF protocols exhausted their energy by round 900 when the network comprised 100 nodes. On the other hand, FQ-UCR continued beyond 2478 rounds, retaining over 50.1% of its energy. Figure 23 and Figure 24 show the residual energy for the 200-node and 400-node networks, respectively. FQ-UCR retained 61.24% and 63.78% of its energy, achieving over 1080 and 1127 rounds of iterations, respectively, whereas the compared protocols depleted their energy sooner. A detailed comparison across different node counts clearly reveals that FQ-UCR outperformed the other two protocols in energy efficiency

#### 5.3.2. Network Lifetime

**Figure 25:**
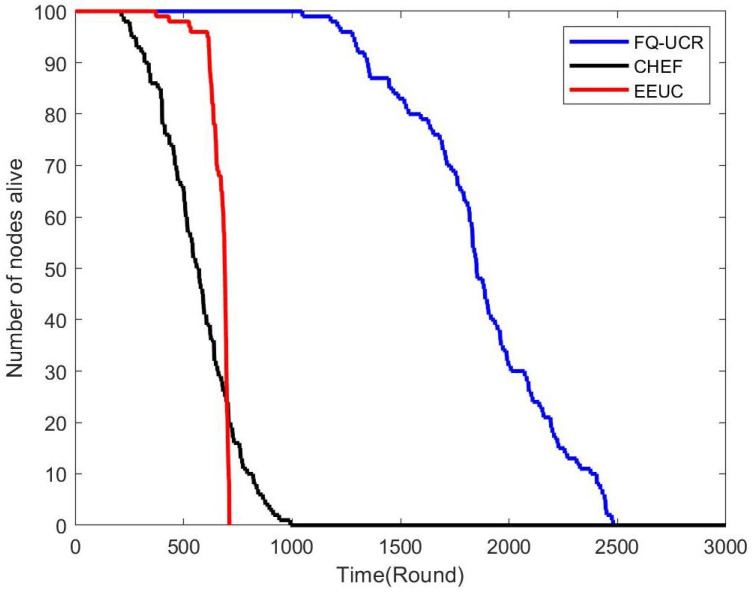
Network lifetime comparison for 100 nodes in Scenario 2.

**Figure 26:**
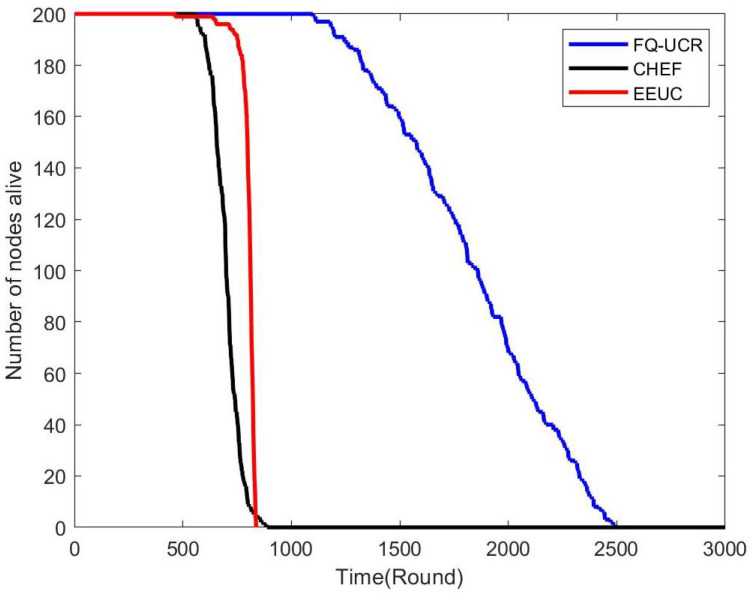
Network lifetime comparison for 200 nodes in Scenario 2.

**Figure 27:**
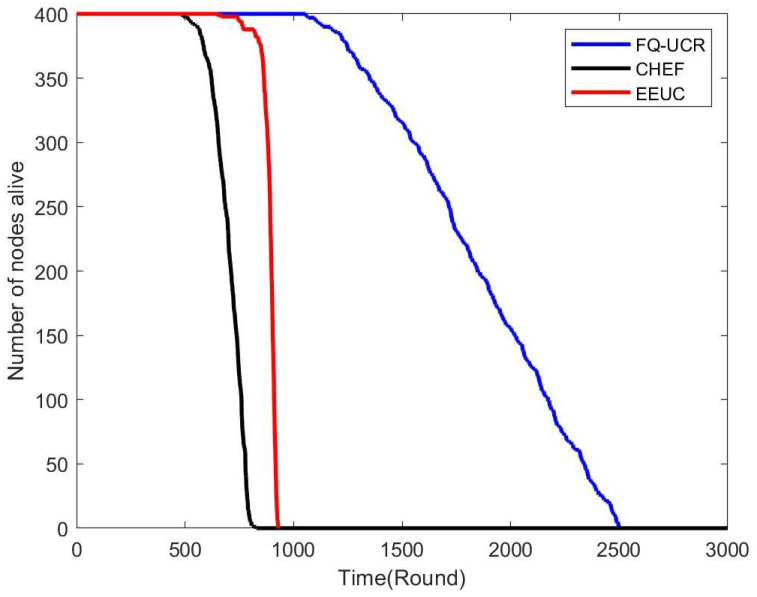
Network lifetime comparison for 400 nodes in Scenario 2.

Figure 25, Figure 26 and Figure 27 present a comparison of lifecycle metrics across the EEUC, CHEF, and FQ-UCR protocols, evaluated with varying node counts. For the 100-node network, both the EEUC and CHEF protocols experienced considerable node depletion around the 900th iteration. In contrast, FQ-UCR exhibited significantly slower node depletion and maintained higher survival rates. Over 175.3% of nodes remained operational after other protocols failed, demonstrating the robustness of FQ-UCR. When scaling up to 200 nodes, the CHEF algorithm showed instability, whereas FQ-UCR lasted over 2492 iterations, outlasting EEUC by 197.73% and CHEF by 181.06%. With 400 nodes, FQ-UCR continued to perform the best, extending the network lifetime by 169.39% compared to EEUC and 201.93% compared to CHEF. These results highlight that FQ-UCR outperformed the comparison protocols, particularly in dense networks with a distant BS, showcasing its superior energy efficiency and resilience.

#### 5.3.3. Stability Period

**Figure 28:**
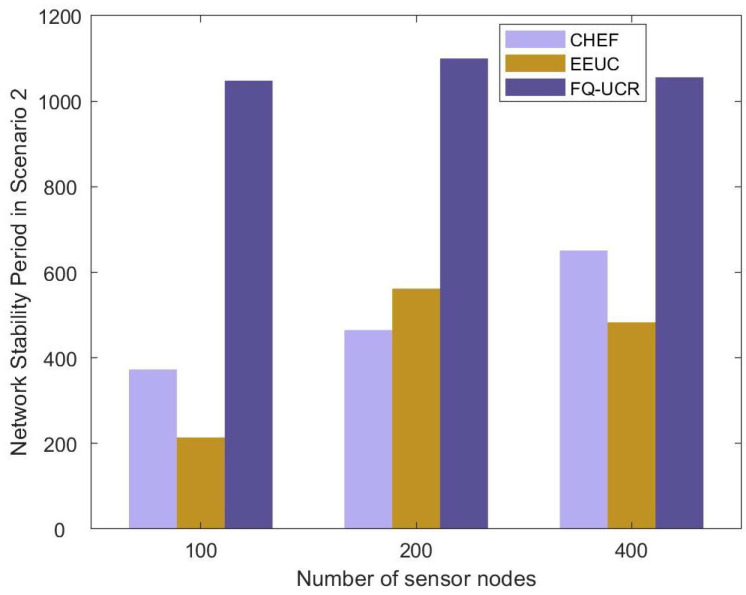
Comparative analysis based on network stability period with BS located at (100, 250).

Table 4 and Figure 28 display the network stability period outcomes for EEUC, CHEF, and FQ-UCR in Scenario 2. With 100 nodes, FQ-UCR’s stability period exceeded that of EEUC and CHEF by 180.9% and 389.7%, respectively. In a network of 200 nodes, FQ-UCR achieved a stability period that was 136.6% longer than that for EEUC and 95.7% longer than that for CHEF. This demonstrates the significant advantage of FQ-UCR, particularly in high-density networks, where it effectively utilized inter-cluster communication and optimized cluster routing strategies to enhance network energy efficiency.

#### 5.3.4. Throughput

**Figure 29:**
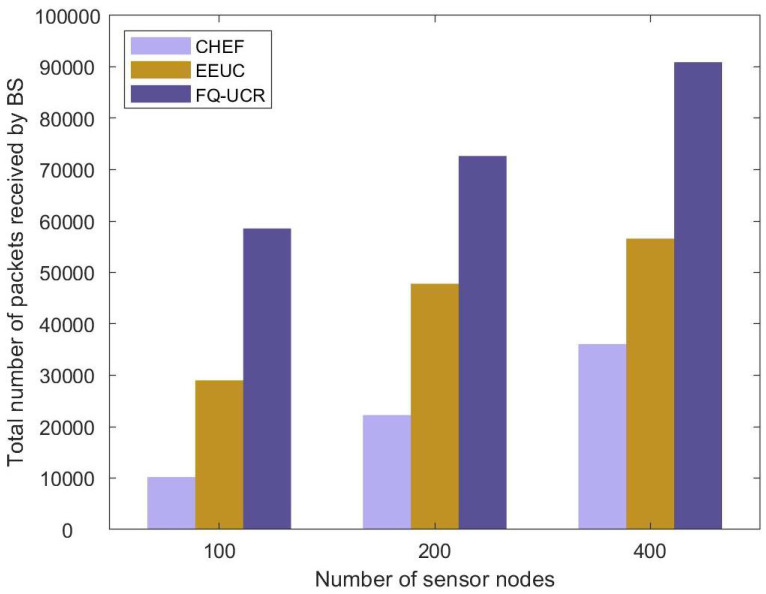
Comparative analysis based on throughput in Scenario 2.

Figure 29 compares the network throughput of the EEUC, CHEF, and FQ-UCR protocols across networks with varying node counts, offering a clear performance evaluation of each protocol. Compared to the other protocols, FQ-UCR achieved a throughput improvement of approximately 101.8% to 151.9% under the same network conditions. The increased throughput resulted from the efficient synergy of the FL and Q-learning methods, which jointly enhanced energy efficiency and extended the network’s lifespan. These elements work together to enhance FQ-UCR’s efficiency in WSNs.

## 6. Conclusions

This paper presents FQ-UCR, an unequal clustering routing algorithm that integrates fuzzy logic and Q-learning to enhance network longevity and effectively address the hotspot issue in WSNs.The proposed FQ-UCR leverages multiple factors as inputs for its FIS for CH selection. Moreover, Q-learning enhances inter-cluster communication to minimize total energy usage.

The simulation results were analyzed across two distinct scenarios: Scenario 1, in which the BS was situated at the network’s center, and Scenario 2, where the BS was positioned outside the network.

In Scenario 1, FQ-UCR demonstrated superior performance, extending the network lifetime by 111.7% compared to CHEF and 228.5% compared to EEUC. Additionally, the stability period saw a substantial enhancement, with increases of 69.3% over CHEF and 120.9% over EEUC.

In Scenario 2, where the BS was located farther from the network, FQ-UCR further excelled by increasing the network lifetime by 202.1% and 169.5% compared to CHEF and EEUC, respectively. Similarly, the stability period was significantly boosted, showing a 118.6% improvement over that of CHEF and a 62.2% improvement over that of EEUC.

As the BS moved farther away, the performance of the CHEF algorithm became increasingly unstable, while FQ-UCR continued to maintain strong performance across transmission efficiency, network stability, and throughput. Future work will focus on refining the fuzzy logic design to further optimize cluster formation, with the ultimate goal of achieving greater energy efficiency and scalability in large-scale WSNs.

## Figures and Tables

**Table 1:** Fuzzy rule base.

No.	Residual Energy	DisToBs	Degree	Centrality	Probability	Radius
1	Low	Near	Few	Close	RatherLow	RatherShort
2	Low	Near	Few	Adequade	Low	VeryShort
3	Low	Near	Few	Far	VeryLow	RatherShort
4	Low	Near	Moderate	Close	Medium–Low	RatherShort
5	Low	Near	Moderate	Adequade	Medium–Low	RatherShort
⋮	⋮	⋮	⋮	⋮	⋮	⋮
77	High	Far	Moderate	Adequade	Medium–High	Medium–Long
78	High	Far	Moderate	Far	RatherHigh	Medium–Long
79	High	Far	Many	Close	High	Long
80	High	Far	Many	Adequade	Medium–High	Long
81	High	Far	Many	Adequade	RatherHigh	Long

**Table 2:** Simulation-related parameters.

Parameter	Scenario 1	Scenario 2
Network size	200 m × 200 m	200 m × 200 m
Number of sensor nodes	100,200,400	100,200,400
Base station location	(100,100) for Scenario 1	(100,250) for Scenario 2
Initial energy	0.5 J	0.5 J
Data packet size	4000 bits	4000 bits
Control packet size	100 bits	100 bits
$E_{elec}$	50 nJ/bit	50 nJ/bit
$\epsilon_{fs}$	10 nJ/bit/$m^{2}$	10 nJ/bit/$m^{2}$
$\epsilon_{mp}$	0.0013 pJ/bit/$m^{4}$	0.0013 pJ/bit/$m^{4}$

**Table 3:** Comparison of network stability period of protocols in Scenario 1.

Number of Sensor Nodes	Protocol	FND (First Node Death)	HND (Half of Nodes’ Death)	LND (Last Node Death)	Stability
100 Nodes	EEUC	380	659	754	380
	CHEF	357	762	1170	357
	FQ-UCR	1376	1922	2477	1376
200 Nodes	EEUC	577	800	827	577
	CHEF	781	980	1136	781
	FQ-UCR	1322	1876	2497	1322
400 Nodes	EEUC	581	897	938	581
	CHEF	841	1079	1326	841
	FQ-UCR	1284	1879	2499	1284

**Table 4:** Comparison of network stability period of protocols in Scenario 2.

Number of Sensor Nodes	Protocol	FND (First Node Death)	HND (Half of Nodes’ Death)	LND (Last Node Death)	Stability
100 Nodes	EEUC	373	690	710	373
	CHEF	214	560	987	214
	FQ-UCR	1048	1851	2478	1048
200 Nodes	EEUC	465	812	837	465
	CHEF	562	700	887	562
	FQ-UCR	1100	1859	2493	1100
400 Nodes	EEUC	651	899	928	651
	CHEF	483	711	828	483
	FQ-UCR	1056	1848	2501	1056

## Data Availability

The original contributions presented in this study are included in the article. Further inquiries can be directed to the corresponding author.
